# Randomized Trial of Hood With or Without Wing Attachment-Assisted Colonoscopy With Linked Color Imaging and Computer-Aided in Adenoma Detection

**DOI:** 10.1055/a-2905-8041

**Published:** 2026-07-09

**Authors:** Kazuya Miyaguchi, Hisashi Matsumoto, Maiko Osawa, Yuki Shiko, Yoshikazu Tsuzuki, Rie Shiomi, Keiji Yamamoto, Yohei Kawasaki, Hiroyuki Imaeda

**Affiliations:** 1 Department of GastroenterologySaitama Medical UniversityMoiroyamaSaitamaJapan; 2 Department of General Internal Medicine 13031 Saitama Medical UniversityIruma-gunSaitamaJapan; 3 Department of Biostatistics, Graduate School of Medicine 13031 Saitama Medical UniversitySaitamaSaitama PrefectureJapan; 4 Department of General Internal Medicine 13031 Saitama Medical UniversityIrumaSaitama PrefectureJapan

**Keywords:** colonoscopy, adenoma detection rate, computer-aided detection, linked color imaging, colorectal cancer screening

## Abstract

**Background and Aim** Computer-aided detection (CADe) combined with linked color imaging (LCI) has shown potential in improving adenoma detection rates (ADRs) during colonoscopies. However, the comparative effectiveness of these supportive methods, based on the type of tip hood, remains unclear. We aimed to compare the ADR between Endo-Wing-assisted colonoscopy with LCI and CADe (ELC) and transparent hood-assisted colonoscopy with LCI and CADe (TLC).

**Methods** This single-center, non-blinded prospective randomized controlled trial enrolled 800 patients who underwent colonoscopy between August 2024 and August 2025. Participants were randomly assigned to either the ELC ( *n* = 400) or TLC group ( *n* = 400). The primary outcome was ADR. Secondary outcomes included the polyp detection rate, adenomas per colonoscopy, and procedural parameters.

**Results** The ELC group demonstrated a significantly higher ADR compared to the TLC group (58.00% vs. 39.75%, *P* < 0.001; relative risk 1.45; 95% CI: 1.25–1.67). This superiority was consistent among expert endoscopists (61.78% vs. 42.17%, *P* < 0.001) as well as trainees (53.14% vs. 35.76%, *P* = 0.002). Furthermore, the ELC group achieved a higher polyp detection rate (61.25% vs. 45.75%, *P* < 0.001) and recorded a greater number of adenomas per colonoscopy (1.17 vs. 0.73, *P* < 0.001). This advantage was particularly pronounced for small adenomas (≤5 mm) and sessile and flat lesions.

**Conclusions** ELC demonstrated a significant improvement in the ADR compared to the TLC approach, particularly for diminutive and sessile adenomas. This superiority was consistent across varying levels of endoscopist experience, indicating broad clinical applicability.

## Introduction

Colorectal cancer remains one of the leading causes of cancer-related mortality worldwide, and colonoscopy plays a central role in its prevention through the detection and removal of adenomatous polyps. 1 The adenoma detection rate (ADR) is an established quality indicator for colonoscopy, with robust evidence demonstrating that each 1% increase in ADR is associated with an approximately 3% reduction in the risk of interval colorectal cancer. 2 3 Nevertheless, a substantial proportion of adenomas—particularly diminutive, flat, or right-sided lesions—continue to be missed during standard colonoscopic examination. 4

To address this limitation, multiple technological innovations have been developed. Linked color imaging (LCI) enhances mucosal color contrast and vascular visibility and has been shown to improve colorectal polyp detection compared with conventional white-light imaging. 5 6 7 8 In parallel, computer-aided detection (CADe) systems provide real-time visual alerts during colonoscopy, and several randomized trials and meta-analyses have demonstrated significant improvements in ADR with CADe-assisted colonoscopy. 9 10 11 12 13 14 15 16 Recent evidence suggests that combining image-enhanced endoscopy with CADe may further improve the detection of subtle lesions. 17

In addition to optical and artificial intelligence-based advances, distal attachment devices designed to improve mucosal exposure—such as transparent caps and Endocuff-assisted colonoscopy—have been evaluated in randomized trials. These devices can flatten colonic folds and improve visualization of blind spots, resulting in modest but variable improvements in ADR. 18 19 20 21 However, most prior studies assessed these devices either in isolation or in combination with a single adjunctive modality, and their incremental value in the contemporary setting—where both image-enhanced endoscopy and CADe are increasingly adopted—remains uncertain.

**Fig. 1:**
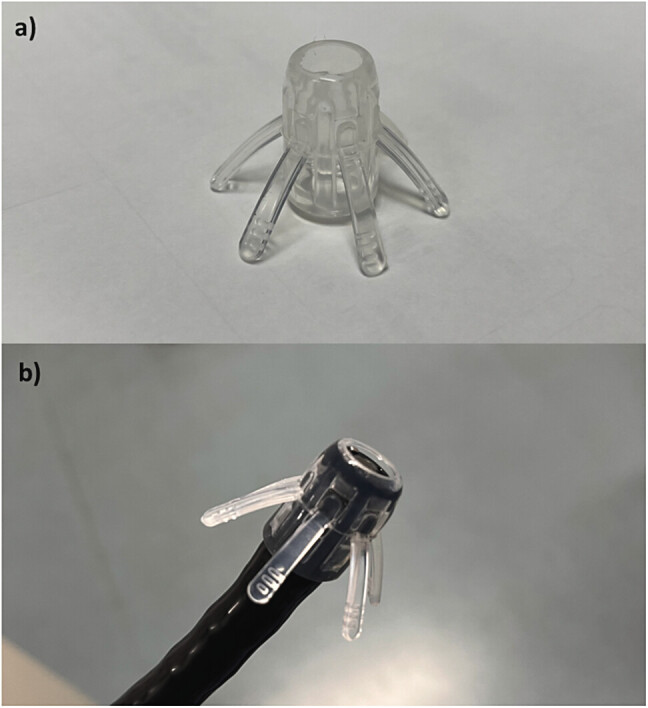
( **a** ) Endo-Wing device. ( **b** ) Image showing attachment of the Endo-Wing to the tip of the endoscope.

The Endo-Wing (Shangxian Minimal Invasive, Inc., Liaoning, China) is a distal attachment device composed of soft, flexible silicone with six expandable wings designed to reduce insertion resistance and enhance mucosal exposure during withdrawal ( Fig. 1 ). Unlike Endocuff-type devices, the Endo-Wing features broader, more pliable arms that may allow more extensive flattening of colonic folds while minimizing mucosal trauma. A recent randomized trial demonstrated superior adenoma detection with the Endo-Wing compared with transparent hood-assisted colonoscopy under conventional imaging conditions. 22 However, whether the structural characteristics of the Endo-Wing confer additional benefits when combined with advanced imaging and CADe has not been systematically evaluated.

Therefore, this prospective randomized controlled trial aimed to compare adenoma detection between Endo-Wing-assisted and transparent hood-assisted colonoscopy, with both groups utilizing LCI and CADe. By employing a contemporary comparator that reflects real-world practice, this study sought to clarify whether optimization of the distal attachment structure can further enhance adenoma detection in the era of image-enhanced endoscopy and artificial intelligence.

## Patients and Methods

### Study Design and Setting

This prospective randomized controlled trial was conducted at Saitama Medical University from August 2024 to August 2025. The study protocol received approval from the Institutional Review Board of Saitama Medical University (Approval No. 2024-041). Written informed consent was obtained from all participants prior to enrollment. The study was registered with the University Hospital Medical Information Network (UMIN: 000054670).

### Participants

Patients aged 20–90 years who were undergoing screening, surveillance, or diagnostic colonoscopy were eligible for enrollment. Diagnostic colonoscopy was defined as a colonoscopy performed for symptoms or abnormal test results in patients without known colorectal neoplasia. Patients with known colorectal lesions prior to the colonoscopy were excluded.

Exclusion criteria included inflammatory bowel disease, polyposis syndrome, prior colectomy, severe comorbidities precluding colonoscopy, and inadequate bowel preparation. All patients were assessed to determine whether they met any of the exclusion criteria prior to randomization, and those meeting any of them were not enrolled in the study.

### Randomization and Interventions

**Fig. 2:**
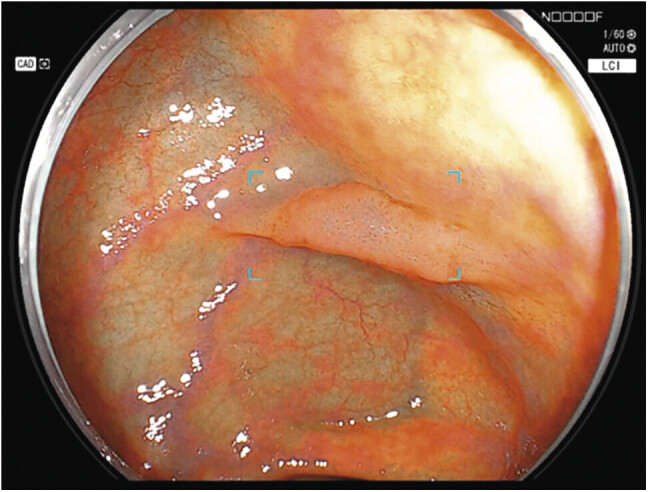
Adenoma detected using endoscopy-assisted linked color imaging (LCI) with computer-aided detection (CADe).

Eligible patients were randomly assigned in a 1:1 ratio to one of the following groups: the ELC group (Endo-Wing-assisted colonoscopy with LCI and CADe) ( Fig. 2 ) and the TLC group (transparent hood-assisted colonoscopy with LCI and CADe).

**Fig. 3:**
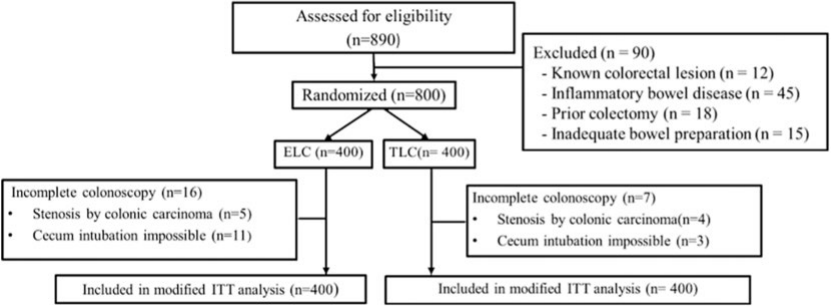
CONSORT flow diagram of patient enrollment and analysis. 29 All exclusion criteria were applied prior to randomization, and no patients were excluded after randomization. Incomplete colonoscopy was defined as failure to achieve cecal intubation or inability to complete the examination due to malignant stenosis. The primary analysis was conducted in a modified intention-to-treat population.

Anonymization was ensured by assigning study-specific codes instead of using personal identifiers such as patient names ( Fig. 3 ). Allocation concealment was achieved through the use of sealed opaque envelopes. The cap device was attached before the insertion of the scope. Group allocation was disclosed by a nurse immediately prior to the initiation of scope withdrawal; thus, endoscopists were aware of the assigned device during withdrawal and were not blinded to group allocation. Patients and pathologists were blinded to group assignment.

Endoscopists were not blinded to group allocation because the distal attachment was visible throughout the procedure. Patients and pathologists were blinded to group assignment.

### Colonoscopy Procedure

Colonoscopy was performed using an Endo-Wing (Shangxian Minimal Invasive, Inc.) or transparent hood (D-201–12704; Olympus Medical Systems, Tokyo, Japan). MoviPrep (EA Pharma Co., Tokyo, Japan) was utilized for bowel preparation. Either scopolamine butylbromide (10 mg) or glucagon (0.5 mg) was administered as an antispasmodic agent. Midazolam (1–4 mg) was administered for conscious sedation only when patients reported abdominal discomfort or pain.

The colonoscope utilized in this study was the EC-L600ZP7 (Fujifilm Co., Tokyo, Japan) in conjunction with the LASEREO 7000 system. This colonoscope provides a field of view of 140°. The CADEYE (EW10EC02; Fujifilm Co.) real-time CADe system was employed throughout the examination.

The quality of bowel preparation was assessed as poor, fair, or good based on the criteria established by Aronchick et al. 23 Bowel preparations rated as fair or good were deemed adequate for analysis, while those rated as poor were excluded. Observations were conducted using white-light imaging during insertion and LCI during withdrawal. When polyps were identified, biopsy or endoscopic resection was performed, followed by histopathological evaluation.

All colonoscopies were conducted by one of five endoscopists: three experts (each having performed over 1000 colonoscopies) and two trainees (each having performed fewer than 1000 colonoscopies). None of the endoscopists exhibited red–green color vision deficiency.

### Histopathology

Resected or biopsied specimens were fixed in 10% formalin and stained with hematoxylin and eosin. Polyps were classified as tubular adenoma, villous adenoma, tubulovillous adenoma, traditional serrated adenoma, sessile serrated lesion, hyperplastic polyp, juvenile polyp, or tubular adenocarcinoma.

### Outcome Measures

Colonic polyps were diagnosed through histopathological examination. The primary outcome measure was the ADR, defined as the proportion of patients with at least one histologically confirmed colorectal neoplastic lesion, including conventional adenomas (tubular, tubulovillous, and villous adenomas), sessile serrated lesions, and traditional serrated adenomas.

Secondary outcomes included the polyp detection rate, adenomas per colonoscopy (APC), APC of diminutive adenomas, APC categorized by colonic segment, adenoma histopathology and morphology, as well as procedural parameters, which encompassed cecal intubation rate, intubation time, withdrawal time, and bowel cleanliness score. Withdrawal time was defined as the time elapsed from cecal intubation to scope removal, excluding therapeutic interventions such as biopsy or polypectomy, but including mucosal inspection and cleaning. Because all detected polyps were resected, this measure was intended to approximate net mucosal inspection time. Withdrawal time was recorded prospectively as part of quality control. The time spent evaluating CADe alerts was included in the withdrawal time and was comparable between the two groups, as identical AI system settings were used.

Adverse events were prospectively assessed in all patients during and after colonoscopy. These included major adverse events such as perforation, as well as minor adverse events including mucosal injury, minor bleeding, and post-procedural discomfort.

ADR and APC were compared between the two groups. Additionally, polyp characteristics, including location, size, and morphology, were compared between expert and trainee endoscopists.

### Statistical Analysis

The sample size was calculated based on a two-sided test for comparison of two independent proportions, assuming an ADR of 55% in the transparent hood-assisted colonoscopy group and 65% in the Endo-Wing-assisted colonoscopy group, corresponding to an anticipated absolute difference of 10%. Using a two-sided significance level (α) of 0.05 and a type II error probability (β) of 0.20 (power = 80%), a minimum sample size of approximately 376 patients per group was required. Therefore, the target enrollment was set at 400 patients per group.

All analyses were performed according to a modified intention-to-treat principle, including all randomized patients. Continuous variables were expressed as means, while categorical variables were presented as counts and percentages. Clinical characteristics were summarized using means ± standard deviation (SD) or proportions. For outcome variables, both means and proportions with corresponding 95% confidence intervals (CIs), as well as between-group differences with 95% CIs, were estimated. Continuous variables were analyzed using Student’s *t* test, whereas categorical variables were assessed using the chi-squared test. The relative risk (RR) with a 95% CI was computed for the primary outcome.

In addition, mixed-effects logistic regression analyses were performed to account for potential clustering by endoscopist, with endoscopist treated as a random effect. Multivariable models were adjusted for age, sex, and examination indication. All statistical tests were two-sided, and statistical significance was established at *P* < 0.05. All statistical analyses, including sample size calculations, were conducted using SAS version 9.4 for Windows (SAS Institute, Cary, NC, USA).

## Results

### Patient Characteristics

A total of 800 patients were randomized, with 400 patients assigned to each group. All randomized patients were included in the primary analysis based on a modified intention-to-treat principle, without any post-randomization exclusion ( Fig. 3 ). The baseline characteristics were well balanced across the groups. The mean age was 66.88 years in the ELC group and 65.42 years in the TLC group. A predominance of male patients was observed in the ELC group (63.25% vs. 51.75%, respectively). The indications for colonoscopy were comparable, with fecal immunochemical test positivity being the most prevalent (37.00% vs. 33.00%). Expert endoscopists performed 56.25% of the procedures in the ELC group and 62.25% in the TLC group ( *P* = 0.084; Table 1 ).

**Table 1:** Clinical characteristics of patients undergoing colonoscopy with linked-color imaging and CADe, with and without assistance from Endo-Wing.

	ELC ( *n* = 400)	TLC ( *n* = 400)	Standardized mean difference (SMD)
Age, mean [SD], years	66.88 [13.47]	65.42 [15.76]	0.10
Sex, *n* (%)			0.23
Male	253 (63.25)	207 (51.75)	
Female	147 (36.75)	193 (48.25)	
Complications/history, *n* (%)			
Prior abdominal surgery	14 (3.50)	8 (2.00)	0.04
Diverticulosis	80 (20.00)	86 (21.50)	0.04
Indication, *n* (%)			
FIT	148 (37.00)	132 (33.00)	0.10
Symptoms	62 (15.50)	69 (17.25)	0.04
Polyp surveillance	86 (21.50)	95 (23.75)	0.04
Screening	88 (22.00)	97 (24.25)	0.04

CADe, computer-aided detection; ELC, Endo-Wing-linked-color imaging with CADe-assisted colonoscopy; LCI, linked-color imaging-assisted colonoscopy; FIT, fecal immunochemical test; TLC, Transparent Hood-LCI-CADe-assisted colonoscopy.

Standardized mean differences (SMDs) were used to assess baseline balance between groups.

### Procedural Characteristics

Cecal intubation rates were high in both groups (96.00% vs. 98.25%, *P* = 0.057), with comparable intubation times (5.42 vs. 5.77 min, *P* = 0.044) and withdrawal times (7.42 vs. 7.34 min, *P* = 0.500). The quality of bowel preparation was excellent, with 99.25% of patients achieving good cleanliness scores using ELC and 98.75% using TLC ( *P* = 0.475; Table 2 ).

**Table 2:** Colonoscopy details of patients undergoing colonoscopy with linked-color imaging and CADe, with and without assistance from Endo-Wing.

	ELC ( *n* = 400)	TLC ( *n* = 400)	Between-group differences ^1^ [95% CI]	*P* value
Cecal intubation rate, *n* (%) [95% CI]	384/400 (96.00) [94.08 to 97.92]	393/400 (98.25) [96.96 to 99.54]	−2.25 [−4.56 to 0.06]	0.057 ^3^
Cecal intubation time, mean [95% CI], min	5.42 [5.22 to 5.62]	5.77 [5.50 to 6.03]	−0.35 [−0.68 to −0.01]	0.044 ^2^
Withdrawal time, mean [95% CI], min	7.42 [7.26 to 7.58]	7.34 [7.15 to 7.52]	0.08 [−0.16 to 0.33]	0.500 ^2^
Cleanliness score, *n* (%) [95% CI]				0.475 ^3^
Good	397/400 (99.25) [98.40 to 100.00]	394/399 (98.75) [97.66 to 99.84]	0.50 [−0.88 to 1.88]	
Fair	3/400 (0.75) [0.00 to 1.60]	5/399 (1.25) [0.16 to 2.34]	−0.50 [−1.88 to 0.88]	
Endoscopist, *n* (%) [95% CI]				0.084 ^3^
Expert	225 (56.25) [51.39 to 61.11]	249 (62.25) [57.50 to 67.00]	−6.00 [−12.80 to 0.80]	
Trainee	175 (43.75) [38.89 to 48.61]	151 (37.75) [33.00 to 42.50]	6.00 [−0.80 to 12.80]	
Adverse events, *n* (%) perforation mucosal injury	0 (0)	0 (0)		

CADe, computer-aided detection; ELC, Endo-Wing-linked-color imaging with CADe-assisted colonoscopy; TLC, Transparent Hood-LCI-CADe-assisted colonoscopy.

^1^ ELC– TLC.

^2^ Student’s *t* test.

^3^ Chi-squared test.

### Primary Outcome: ADR

The ELC group demonstrated a significantly higher ADR compared to the TLC group, with rates of 58.00% and 39.75%, respectively, resulting in a difference of 18.25% (95% CI: 11.44–25.06%, *P* < 0.001). The RR of adenoma detection with ELC was 1.45 (95% CI: 1.25–1.67).

This superiority was consistently observed across various levels of endoscopist experience: (1) Expert endoscopists: 61.78% versus 42.17%, resulting in a difference of 19.61% (95% CI: 10.78–28.44%, *P* < 0.001; RR 1.45; 95% CI: 1.22–1.73); (2) Trainee endoscopists: 53.14% versus 35.76%, leading to a difference of 17.38% (95% CI: 6.75–28.02%, *P* = 0.002; RR: 1.48; 95% CI: 1.15–1.90). Multivariable logistic regression analysis adjusting for age, sex, examination indication, and endoscopist demonstrated that ELC remained independently associated with a higher ADR compared with TLC (adjusted odds ratio [OR]: 2.27, 95% CI: 1.66–3.10; *P* < 0.001; Table 3 and **Supplementary Table 1** ). Similar results were obtained in mixed-effects logistic regression analyses accounting for endoscopist-level clustering ( **Supplementary Table 2** ). Per-protocol analyses excluding incomplete colonoscopy examinations yielded results consistent with the primary analysis ( **Supplementary Table 3** ).

**Table 3:** Adenoma detection with linked-color imaging and CADe, with and without assistance from Endo-Wing.

	ELC ( *n* = 400)	TLC ( *n* = 400)	Between-group differences ^1^ [95% CI]	*P* value ^2^	Adjusted OR (95% CI)*
ADR, n (%) [95% CI]	232 (58.00) [53.16 to 62.84]	159 (39.75) [34.95 to 44.55]	18.25 [11.44 to 25.06]	<0.001	2.27 (1.66–3.10)
ADR in experts	139/225 (61.78) [55.43 to 68.13]	105/249 (42.17) [36.03 to 48.30]	19.61 [10.78 to 28.44]	<0.001	
ADR in trainees	93/175 (53.14) [45.75 to 60.54]	54/151 (35.76) [28.12 to 43.41]	17.38 [6.75 to 28.02]	0.002	
Relative risk [95% CI] (vs. TLC)					
ADR	1.45 [1.25 to 1.67]				
ADR in experts	1.45 [1.22 to 1.73]				
ADR in trainees	1.48 [1.15 to 1.90]				

CADe, computer-aided detection; ELC, Endo-Wing-linked-color imaging with CADe-assisted colonoscopy; TLC, Transparent Hood-LCI-CADe-assisted colonoscopy; ADR, adenoma detection rate.

^1^ ELC – TLC.

^2^ Chi-squared test.

*Adjusted for age, sex, examination indication, and endoscopist-level clustering using mixed-effects logistic regression analysis with the endoscopist treated as a random effect.

### Secondary Outcomes ( Table 4 )

**Table 4:** Adenoma detection with linked-color imaging and CADe, with and without assistance from Endo-Wing.

	ELC ( *n* = 400)	TLC ( *n* = 400)	Between-group differences ^1^ [95% CI]	*P* value ^2^
PDR, n (%) [95% CI]	245/400 (61.25) [56.48 to 66.02]	183/400 (45.75) [40.87 to 50.63]	15.50 [8.67 to 22.33]	<0.001 ^3^
APC, mean [95% CI]	1.17 [1.03 to 1.31]	0.73 [0.62 to 0.85]	0.44 [0.26 to 0.62]	<0.001 ^2^
Size, mean [95% CI]				
≤5 mm	0.77 [0.66 to 0.88]	0.45 [0.36 to 0.53]	0.33 [0.19 to 0.47]	<0.001 ^2^
6–9 mm	0.24 [0.18 to 0.29]	0.17 [0.12 to 0.22]	0.07 [−0.01 to 0.14]	0.094 ^2^
≥10 mm	0.16 [0.12 to 0.21]	0.12 [0.08 to 0.16]	0.04 [−0.01 to 0.10]	0.147 ^2^
Morphology, mean [95% CI]				
Sessile	0.79 [0.68 to 0.90]	0.43 [0.35 to 0.51]	0.36 [0.22 to 0.50]	<0.001 ^2^
Nonpolypoid	0.11 [0.07 to 0.15]	0.03 [0.01 to 0.05]	0.08 [0.04 to 0.13]	<0.001 ^2^
Pedunculated	0.28 [0.21 to 0.34]	0.27 [0.20 to 0.34]	0.01 [−0.09 to 0.10]	0.916 ^2^
Colonic segment, mean [95% CI]				
Cecum	0.10 [0.06 to 0.13]	0.06 [0.03 to 0.08]	0.04 [0.00 to 0.08]	0.046 ^2^
Ascending colon	0.29 [0.23 to 0.35]	0.15 [0.11 to 0.20]	0.14 [0.06 to 0.21]	<0.001 ^2^
Transverse colon	0.28 [0.22 to 0.34]	0.19 [0.14 to 0.24]	0.09 [0.01 to 0.17]	0.026 ^2^
Descending colon	0.08 [0.05 to 0.11]	0.04 [0.02 to 0.06]	0.04 [0.00 to 0.07]	0.038 ^2^
Sigmoid colon	0.35 [0.29 to 0.42]	0.26 [0.20 to 0.32]	0.09 [0.00 to 0.18]	0.046 ^2^
Rectum	0.08 [0.05 to 0.10]	0.04 [0.02 to 0.06]	0.04 [0.00 to 0.07]	0.042 ^2^
Histopathology, mean [95% CI]				
Tubular adenoma	0.86 [0.74 to 0.97]	0.52 [0.44 to 0.61]	0.34 [0.19 to 0.48]	<0.001 ^2^
Tubulovillous adenoma	0.05 [0.03 to 0.07]	0.05 [0.02 to 0.07]	0.00 [−0.03 to 0.04]	0.884 ^2^
SSL	0.03 [0.02 to 0.05]	0.02 [0.01 to 0.03]	0.01 [−0.01 to 0.03]	0.269 ^2^
Villous adenoma	0.04 [0.02 to 0.05]	0.02 [0.01 to 0.03]	0.02 [−0.01 to 0.04]	0.195 ^2^
Traditional serrated adenoma	0.06 [0.03 to 0.09]	0.03 [0.01 to 0.04]	0.04 [0.00 to 0.07]	0.054 ^2^
High-grade dysplasia	0.09 [0.06 to 0.12]	0.06 [0.04 to 0.09]	0.03 [−0.01 to 0.07]	0.131 ^2^
Submucosal adenocarcinoma	0.02 [0.00 to 0.03]	0.01 [0.00 to 0.02]	0.00 [−0.01 to 0.02]	0.765 ^2^
Advanced carcinoma	0.04 [0.02 to 0.05]	0.03 [0.01 to 0.04]	0.01 [−0.02 to 0.03]	0.543 ^2^

CADe, computer-aided detection; ELC, Endo-Wing-linked-color imaging with CADe-assisted colonoscopy; TLC, Transparent Hood-LCI-CADe-assisted colonoscopy; LCI, linked-color imaging-assisted colonoscopy; APC, adenomas per colonoscopy; SSL, sessile serrated lesion.

^1^ ELC– TLC.

^2^ Student’s *t* test.

^3^ Chi-squared test.

#### Polyp Detection Rate

The ELC group demonstrated a significantly higher polyp detection rate (61.25% vs. 45.75%, difference 15.50%, 95% CI: 8.67–22.33%, *P* < 0.001) and a greater number of APC (1.17 vs. 0.73, difference 0.44, 95% CI: 0.26–0.62, *P* < 0.001) when compared to the TLC group.

#### Adenoma Characteristics by Size

The benefit of ELC was most pronounced for diminutive adenomas measuring ≤5 mm (0.77 vs. 0.45 per colonoscopy, *P* < 0.001). Smaller differences were observed for larger lesions, including those measuring 6–9 mm (0.24 vs. 0.17, *P* = 0.094) and ≥10 mm (0.16 vs. 0.12, *P* = 0.147).

#### Adenoma Morphology

ELC demonstrated a significantly higher detection rate of sessile adenomas (0.79 vs. 0.43 per colonoscopy, *P* < 0.001) and nonpolypoid lesions (0.11 vs. 0.03, *P* < 0.001), while displaying comparable performance for pedunculated lesions (0.28 vs. 0.27, *P* = 0.916).

#### Anatomical Distribution

Enhanced detection using ELC was observed across all colonic segments, with the most significant difference noted in the ascending colon (0.29 vs. 0.15, *P* < 0.001).

#### Histopathological Findings

Tubular adenomas represented the majority of the identified lesions, with ELC demonstrating superior detection rates (0.86 vs. 0.52 per colonoscopy, *P* < 0.001). While the detection rates for advanced lesions, including high-grade dysplasia and traditional serrated adenomas, showed numerical advantages for ELC, statistical significance was not achieved due to their low prevalence.

### Safety and Tolerability

No procedure-related adverse events, including perforation, delayed bleeding, or mucosal injury, were observed in either group. Although incomplete cecal intubation occurred more frequently in the ELC group than in the TLC group, no clear device-related cause was identified. Both technologies were well tolerated by the patients during the study procedure ( Table 2 ).

## Discussion

In this randomized controlled trial, Endo-Wing-assisted colonoscopy combined with LCI and CADe significantly improved adenoma detection compared with transparent hood-assisted colonoscopy using the same imaging and AI support. The absolute increase in ADR in ELC was consistently observed across endoscopist experience levels and was most pronounced for diminutive (≤5 mm), sessile, and nonpolypoid adenomas, as well as lesions located in the right colon.

Several randomized trials have previously evaluated the combined use of mucosal exposure devices and CADe. The CERTAIN trial reported a modest incremental benefit when Endocuff Vision was added to CADe-assisted colonoscopy, while other studies demonstrated limited but statistically significant improvements when Endocuff was combined with artificial intelligence-based detection. 21 24 25 In contrast to these studies, the present trial evaluated the Endo-Wing—a device with distinct structural characteristics—under fully contemporary conditions in which both LCI and CADe were applied in both study arms. This design allowed us to isolate the effect of distal attachment structure rather than simply confirming the benefit of adding a device to CADe alone.

The magnitude of ADR improvement observed in this study exceeds that reported in most prior trials of mucosal exposure devices or image-enhanced endoscopy alone. Meta-analyses of CADe-assisted colonoscopy have generally reported relative ADR improvements of approximately 20–30%, whereas LCI alone has been associated with more modest gains of around 10%. 7 8 9 10 11 12 13 16 Similarly, cap-assisted or Endocuff-assisted colonoscopy has yielded absolute ADR increases ranging from 5% to 15%, with considerable heterogeneity across studies. 18 19 20 21 The ADR of 58% achieved in the Endo-Wing group in the present trial exceeds both established minimum quality benchmarks and recently proposed aspirational targets. 26 27

The enhanced performance of Endo-Wing-assisted colonoscopy was particularly evident for diminutive, sessile, and right-sided lesions, which are more likely to be missed during standard colonoscopy because of limited mucosal visualization in the right colon and may contribute to interval colorectal cancer. 3 4 28 Although diminutive adenomas have limited immediate malignant potential, their detection contributes to achieving a “clean colon” and may reduce cumulative cancer risk. Therefore, improved detection of these lesions may have clinically meaningful implications beyond their use as short-term quality indicators. In contrast, no significant difference was observed for larger or advanced lesions, likely reflecting their lower prevalence and greater conspicuity.

Importantly, these improvements were achieved without prolongation of withdrawal time or compromise of procedural efficiency. Although a small difference in insertion time was observed, the absolute magnitude was minimal and unlikely to be clinically meaningful. No device-related adverse events were identified, supporting the safety of Endo-Wing under the conditions of this study. However, incomplete cecal intubation occurred more frequently in the ELC group than in the TLC group. Although Endo-Wing is designed to reduce insertion resistance through its flexible wings, the presence of the device at the tip of the endoscope may theoretically affect maneuverability in certain situations. In the present study, no definitive device-related cause was identified; however, a potential contribution to insertion difficulty cannot be completely excluded. Several factors should be considered when interpreting the magnitude of the observed effect. First, this was a single-center study conducted under highly standardized conditions with excellent bowel preparation in most participants, which may limit generalizability to routine clinical practice. In addition, the study utilized a specific Fujifilm LCI platform and CADe system. Although the mechanical advantage of the Endo-Wing is likely to be broadly applicable, potential interactions with different imaging technologies and CADe platforms cannot be excluded, and external validation in other settings is warranted. Second, the control group utilized CADe and LCI in combination with a transparent hood rather than CADe alone, which likely represents a higher-performance comparator than that used in some prior studies. Third, the Endo-Wing’s broader and more flexible contact surface compared with Endocuff-type devices may have contributed to more effective fold flattening during withdrawal. Together, these factors may have amplified the observed effect size and should be considered when extrapolating these findings to other settings.

This study has several limitations. Endoscopists were not blinded to the assigned distal attachment, as the device was visible throughout the procedure, which may have introduced performance bias. However, withdrawal times were comparable between groups, suggesting similar inspection effort. Although some imbalance in sex and FIT positivity distribution was observed between groups, additional adjusted analyses accounting for baseline characteristics and endoscopist-level clustering yielded results consistent with the primary analysis ( **Supplementary Table 1** ). Long-term outcomes, such as interval colorectal cancer incidence, were not assessed. Additionally, because the Endo-Wing was evaluated only in combination with LCI and CADe, its independent mechanical effect under conventional white-light imaging could not be directly assessed, and the absence of a high-definition white-light endoscopy control arm limits direct comparison with previous studies of distal attachment devices. Potential issues such as subtle mucosal microtrauma, operator fatigue, and maneuverability during prolonged procedures were not formally evaluated and warrant further investigation.

In conclusion, Endo-Wing-assisted colonoscopy combined with LCI and CADe significantly improved adenoma detection compared with transparent hood-assisted colonoscopy using the same advanced imaging and AI support. This benefit was consistent across endoscopist experience levels and was most pronounced for diminutive, sessile, and right-sided adenomas. These findings suggest that optimization of the distal attachment structure may further enhance the effectiveness of advanced imaging and artificial intelligence-assisted colonoscopy, supporting a multimodal approach to improving colonoscopy quality.

## Supplementary Material

Ergänzendes Material Supplementary Material
