# Supplementary material for: Randomized Trial of Hood With or Without Wing Attachment-Assisted Colonoscopy With Linked Color Imaging and Computer-Aided in Adenoma Detection
Source: Endosc Int Open. 2026 Jul 9;14:a29058041. doi: 10.1055/a-2905-8041 (PMC13358689; doi:10.1055/a-2905-8041)
Supplement: Supplementary file 1 — Ergänzendes Material [file 10-1055-a-2905-8041_29061876.pdf]

Supplementary Table 1.

Multivariable logistic regression analysis for adenoma detection rate

Variable	Adjusted OR	95% CI	P value
ELC vs TLC	2.27	1.66–3.10	<0.001
Age (per year)	1.03	1.02–1.04	<0.001
Female sex (vs male)	0.52	0.38–0.71	<0.001
Symptoms vs FIT	1.32	0.85–2.05	0.646
Surveillance vs FIT	1.65	1.10–2.48	0.032
Screening vs FIT	1.04	0.70–1.55	0.231

Multivariable logistic regression analysis adjusted for age, sex, examination indication, and endoscopist.

ELC, Endo-Wing-linked-color imaging with CADe-assisted colonoscopy; TLC, transparent hood-assisted colonoscopy with linked-color imaging and CADe.

Supplementary Table 2.

Mixed-effects logistic regression analysis for ADR with endoscopist treated as a random effect

Variable	Adjusted OR	95% CI	P value
ELC vs TLC	2.19	1.59–2.85	<0.001

ELC, Endo-Wing-linked-color imaging with CADe-assisted colonoscopy.

TLC, Transparent Hood-LCI-CADe-assisted colonoscopy.

Supplementary Table 3

Per-protocol population excluding incomplete colonoscopy examinations

	ELC (n = 384)	TLC (n = 393)	Between-group differences[1] [95%CI]	*P* value[2]
ADR, n (%) [95%CI]	224 (58.33) [53.40 to 63.26]	156 (39.69) [34.86 to 44.53]	18.64 [11.73 to 25.55]	<.001
ADR in experts	136/216 (62.96) [56.52 to 69.40]	102/244 (41.80) [35.61 to 47.99]	21.16 [12.23 to 30.09]	<.001
ADR in trainees	88/168 (52.38) [44.83 to 59.93]	54/149 (36.24) [28.52 to 43.96]	16.14 [5.34 to 26.94]	0.004
Relative risk [95%CI] (vs. TLC)				
ADR	1.45 [1.26 to 1.68]			
ADR in experts	1.49 [1.25 to 1.78]			
ADR in trainees	1.43 [1.11 to 1.83]			

CADe, computer-aided detection

ELC, Endo-Wing-linked-color imaging with CADe-assisted colonoscopy.

TLC, Transparent Hood-LCI-CADe-assisted colonoscopy; ADR, adenoma detection rate.

[1]ELC – TLC.

[2]Chi-squared test.
